# Tamsulosin attenuates high glucose- induced injury in glomerular endothelial cells

**DOI:** 10.1080/21655979.2021.1955527

**Published:** 2021-08-17

**Authors:** Lin Sun, Chengmin Sun, Shibo Zhou, Lan Zhang, Wenping Hu

**Affiliations:** a Department of Pharmacy Intravenous Admixture Service, The Fourth Affiliated Hospital of Harbin Medical University, Harbin, Heilongjiang, China; b Department of Traditional Chinese Medicine, The Fourth Affiliated Hospital of Harbin Medical University, Harbin, Heilongjiang, China; c Inpatient Department Central Pharmacy, The Fourth Affiliated Hospital of Harbin Medical University, Harbin, Heilongjiang, China; d Department of Cardiology, The Fourth Affiliated Hospital of Harbin Medical University, Harbin, Heilongjiang, China; e Department of Nephrology, The Fourth Affiliated Hospital of Harbin Medical University, Harbin, Heilongjiang, China

**Keywords:** Diabetic nephropathy, tamsulosin, oxidative stress, inflammation, fibrosis, nf-κB

## Abstract

Diabetic nephropathy (DN) is a common complication of diabetes. Tamsulosin is a selective α1-AR antagonist. α1-AR is expressed widely in kidney tissues and has displayed its various physiological functions. However, whether Tamsulosin has affects DN is unknown. To our knowledge, this is the first time it has been examined whether Tamsulosin possesses a beneficial effect in high glucose-challenged glomerular endothelial cells (GECs). Firstly, we found that Tamsulosin reduced high glucose-induced expressions of TNF-α, IL-6, and IL-8. Secondly, Tamsulosin alleviated high glucose-induced expressions of MMP-2 and MMP-9. Thirdly, Tamsulosin inhibited the expressions of VCAM-1 and ICAM-1. Importantly, our results indicate that Tamsulosin inhibited high glucose-induced expressions of fibrosis factors such as Col-1 and TGF-β1. Additionally, we found that Tamsulosin ameliorated oxidative stress via reducing the generation of ROS and preventing the activation of p38. Mechanistically, we found that Tamsulosin attenuated high glucose-induced activation of NF-κB. Based on these findings, we conclude that Tamsulosin could attenuate high glucose-induced injury in GECs through alleviating oxidative stress and inflammatory response.

## Graphical abstract

**Figure:**
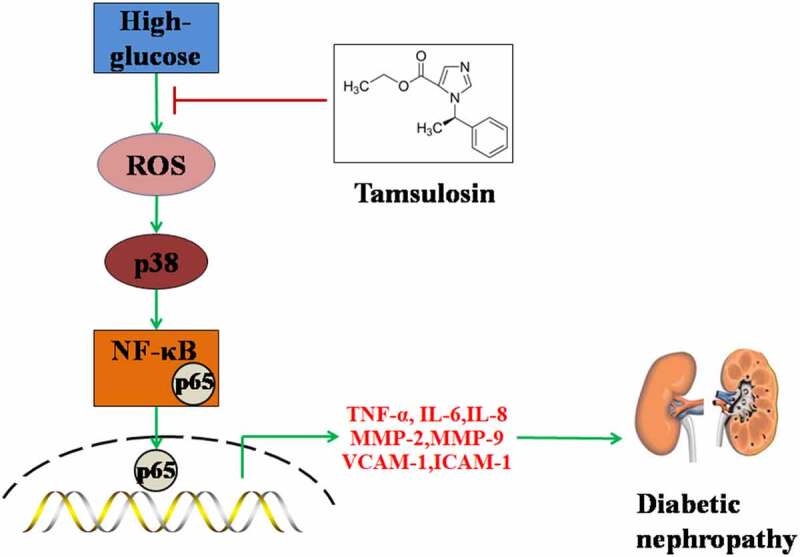

## Introduction

Diabetic nephropathy (DN) is commonly observed in diabetic patients. Approximately 30–40% of diabetic patients develop kidney dysfunction [1]. Owing to the high prevalence of diabetes, DN has been considered as the major risk factor of end-stage renal disease (ESRD) [2]. Thus, it is of great importance to study the potential treatments for DN. DN is characterized by structural changes to the glomerulus, including diffuse basement membrane thickening, glomerular hypertrophy, and excess accumulation of extracellular matrix, resulting in glomerulosclerosis and tubulointerstitial fibrosis. Chronic inflammation and renal interstitial fibrosis are associated with the progression of DN, ultimately leading to renal failure [3,4]. Microalbuminuria is the hallmark of glomerular endothelial cells (GECs) injury and is also a common clinical symptom in the early stage of DN [5,6]. GECs injury plays an important part in oxidative stress, chronic inflammation, and renal interstitial fibrosis, which contribute to the development of DN [7]. High glucose is well known to be one of the most important risk factors contributing to the pathogenesis of DN by inducing the dysfunction of GECs [8]. High glucose is reported to cause oxidative stress by promoting ROS overproduction in mitochondria [9,10]. Additionally, excessive generations of TNF-α, IL-6, and IL-8 have been considered as a prerequisite for microvasculature endothelial dysfunction under high-glucose conditions. Chronic inflammation could cause tissue damage and fibrosis in numerous diseases, including DN. For example, fibrosis factors such as Col-1 and TGF-β1 have been involved in high glucose-induced renal interstitial fibrosis. Therefore, amelioration of high glucose-induced damage in GECs is important for the treatment of DN.

Tamsulosin is a selective α1-AR antagonist. α1-AR is a subtype of adrenoreceptors (ARs) that belong to the G protein-coupled receptors (GPCRs) superfamily. α1-AR is widely distributed in the kidney and regulates some important renal functions such as metabolism and tubule function via several signaling pathways, including p38 mitogen-activated protein kinase (MAPK) [11,12]. In recent years, Tamsulosin has been considered a potent agent used in adjunctive medical expulsive therapy after extracorporeal shock wave lithotripsy [13]. A previous study identified that the activation of α1-AR augmented the release of IL-1β, a key pro-inflammatory cytokine in various inflammatory responses. Meanwhile, another study showed that α1-AR could promote renal fibrosis in TGF-β1-challenged HK-2 cells and a mouse model. As inflammation and renal interstitial fibrosis are key steps in the development of DN, these findings indicated that the inhibition of α1-AR might have protective benefits in DN. However, whether Tamsulosin has any anti-inflammatory capacity in the development of DN is still unknown. In this study, we aim to study the effects of Tamsulosin, the selective antagonist of α1-AR, on high glucose-induced injury in GECs.

## 2. Materials and methods

### 2.1 Cell culture and treatment

Human glomerular endothelial cells (GECs) were purchased from ScienCell (Carsbad, USA) and cultured in an endothelial growth medium (Lonza, Walkersville, USA), with 10% FBS and 1% antibiotics. The cells were cultured in a T-75 cell culture flask which was coated with human gelatin. For experiments, cells were cultured in 6-well or 12-well plates and stimulated with high glucose (25 mM) [14] or Tamsulosin (25, and 50 nM) [15] for 24 hours. Cells treated with 5 mM glucose were used as normal control.

### 2.2. Real-time PCR

Total RNA from GECs was isolated using TRIzol (Invitrogen, USA, Cat#15,596,026) according to the instructions of the product, and a DNAse (Invitrogen, USA, Cat#EN0521) treatment was included in the end. Total RNA concentrations were measured using Thermo Scientific NanoDrop machine. 2 μg total RNA was transcribed to cDNA using TaqMan MicroRNA Reverse Transcription Kit (Thermo Fisher Scientific, USA, Cat#4,366,596). cDNA was synthesized and subjected to qPCR with SYBR green mix (Cat#4,366,596, Thermo Fisher Scientific, USA) on a LightCycler 480 System qPCR machine. The expression of target genes was normalized to β-actin using the 2 ^– ΔΔCt^ method.

### 2.3. ELISA assay

The secretions of proteins were determined using ELISA assays. Following ELISA kits from Solarbio life sciences were used in this study: MMP-2 (Cat#SEKH-0253), MMP-9 (Cat#SEKH-0257), VCAM-1 (Cat#SEKH-0055), ICAM-1(Cat#SEKH-0053), COL1α1 (Cat#EH0958), and TGF-β1 (Cat#EH0287). The ELISA was performed according to the protocol from the manufacturer. The expression level was measured at the wavelength of 490 nm using a microplate reader.

### 2.4. Mitochondrial ROS measurement

5 × 10^4^/well GECs were planted in 12-well plates. After demanded treatments, the cells were washed and probed with 5 µm MitoSOX red for 10 minutes. The fluorescence was then captured using a fluorescent microscope (Excitation wavelength: 385 nm; Emission wavelength 430 nm). Fluorescent intensity was measured using the software Image J.

### 2.5. Western blot analysis

Cells were lysed using cell lysis buffer with Protease/Phosphatase Inhibitor Cocktail (Cell Signaling Technologies, USA). Protein concentrations were measured using bicinchoninic acid (BCA) protein assay. 20 µg protein was separated by 12% SDS-PAGE. The proteins were then transferred to PVDF membranes. The membranes were blocked with 5% fat-free milk diluted in TBS-T for an hour and then incubated with phosphorylated p38 (#4511, Cell Signaling Technologies, USA), p38 (#8690, Cell Signaling Technologies, USA), NF-κB p65 (#8242, Cell Signaling Technologies, USA), β-actin (#3700, Cell Signaling Technologies, USA) or Lamin B1 (#17,416, Cell Signaling Technologies, USA) primary antibodies at 4 °C overnights and HRP-conjugated secondary antibody for 1 hour. Blots were visualized using ECL [16].

### 2.6. Cell viability assessment

Cell viability of GECs was measured using the CCK-8 kit. Briefly, 100 μL cell suspension was added to a 96-well plate. After treatment, 10 μL of the CCK-8 solution was added to each well of the plate. After incubation for 2 hours at 37 °C, absorbance was recorded at 450 nM to reflect cell viability.

### 2.7. Determination of NO levels

GECs were seeded in a 96-well plate at 4 × 10^3^ per well. After treatment, the levels of NO were measured using a NO detection kit (Beyotime, Beijing, China). Briefly, 50 μL cell culture supernatant or standard sample was mixed with an equal amount of Griess Reagent I and Griess Reagent II. Absorbance at 540 nm was measured to index the levels of NO.

### 2.8. Statistical analysis

All experiments were performed more than three times. Results are shown as mean ± standard deviation (SD). Differences were analyzed using ANOVA. A p-value less than 0.05 was considered statistically significant.

## 3. Results

Using a high glucose-stimulated human GECs model, we investigated the effects of Tamsulosin on the generation of ROS, expressions of pro-inflammatory cytokines, MMPs, adhesion molecules, and fibrosis factors. We found that Tamsulosin attenuated the inflammatory response and fibrosis in human GECs via downregulating the activation of NF-κB.

## 3.1. Tamsulosin attenuated high glucose-induced expression of pro-inflammatory cytokines in human GECs.

**Figure 1.**
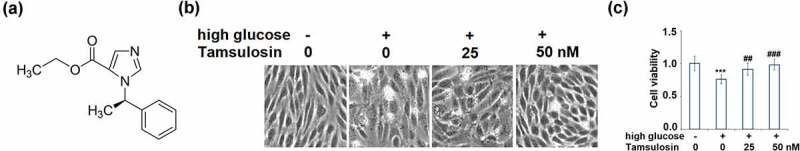
The effects of Tamsulosin on the morphology of high glucose-stimulated human GECs. (a). The structure of Tamsulosin; (b). Cells were stimulated with high glucose (25 mM) in the presence or absence of Tamsulosin (25, 50 nM) for 24 hours. Morphology of GECs; (c). Cell viability of GECs (***, P < 0.005 vs. vehicle group; ##, ###, P < 0.05, 0.01 vs. high glucose group, N = 6)

**Figure 2.**
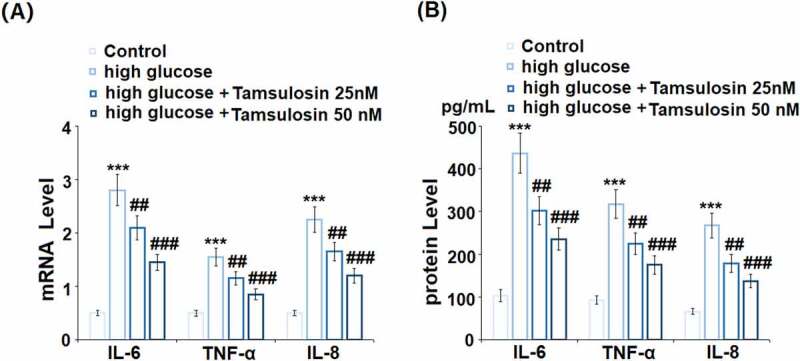
Tamsulosin attenuated high glucose-induced expression of pro-inflammatory cytokines in human glomerular endothelial cells (GECs). (a). mRNA of IL-6, TNF-α, IL-8; (b). Secretions of IL-6, TNF-α, and IL-8 (***, P < 0.005 vs. vehicle group; ##, ###, P < 0.05, 0.01 vs. high glucose group, N = 5)

The molecular structure of Tamsulosin is shown in Figure 1a. The effects of Tamsulosin on the morphology of high glucose-stimulated human GECs are shown in Figure 1b. Interestingly, results in Figure 1c demonstrate that exposure to high glucose reduced the cell viability of human GECs, which was rescued by Tamsulosin. The expressions and production of pro-inflammatory cytokines were measured. High glucose significantly increased the mRNA levels of IL-6, TNF-α, and IL-8 to 5.6-, 3.1 – and 4.5 – fold, respectively. These were then decreased to 4.2-, 2.3 – and 3.3 – fold by 25 nM Tamsulosin, respectively. Furthermore, 50 nM Tamsulosin reduced the expressions of these three cytokines to 2.9-, 1.7 – and 2.4 – fold, respectfully. Similarly, ELISA results in Figure 2b show that 25 and 50 nM Tamsulosin also inhibited the expressions of IL-6, TNF-α, and IL-8 at the protein level, compared with the significant increase induced by high glucose.

### 3.2 Tamsulosin reduced MMP-2 and MMP-9

**Figure 3.**
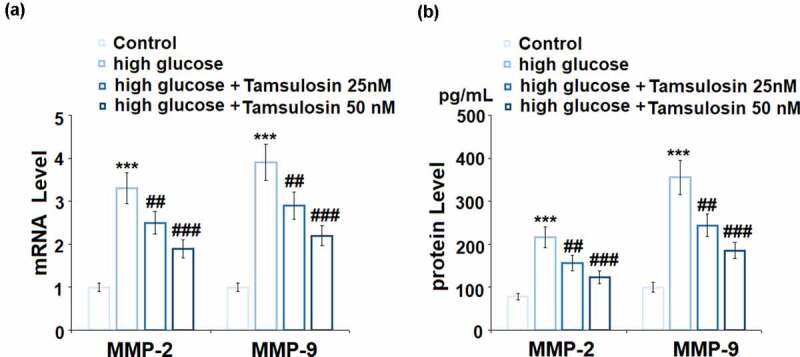
Tamsulosin alleviated high glucose-induced expressions of MMP-2 and MMP-9. (a). mRNA of MMP-2 and MMP-9; (b). Protein of MMP-2 and MMP-9 (***, P < 0.005 vs. vehicle group; ##, ###, P < 0.05, 0.01 vs. high glucose group, N = 4–5)

We further detected the effect of Tamsulosin on the expressions of MMP-2 and MMP-9. As shown in Figure 3a, treatment with high glucose-induced a 3.3 – and 3.9 – fold increase in the mRNA levels of MMP-2 and MMP-9, respectively. However, 25 and 50 nM Tamsulosin decreased these levels to 2.5 – and 1.9 – fold of MMP-2, and 2.9 – and 2.2 – fold of MMP-9, respectively. The same doses of Tamsulosin exhibited similar effects on the protein levels of MMP-2 and MMP-9. As shown in Figure 3b, high glucose elevated the protein level of MMP-2 in GECs from 78.3 to 216.5 mg/mL, which was reduced to 156.8 and 123.4 pg/mL by 25 and 50 nM Tamsulosin, respectively. The protein level of MMP-9 was increased to 355.2 pg/mL by high glucose stimulation and reduced to 243.9 and 185.5 pg/mL by the same doses of Tamsulosin, respectively.

### 3.3 Tamsulosin inhibited the expressions of VCAM-1 and ICAM-1

**Figure 4.**
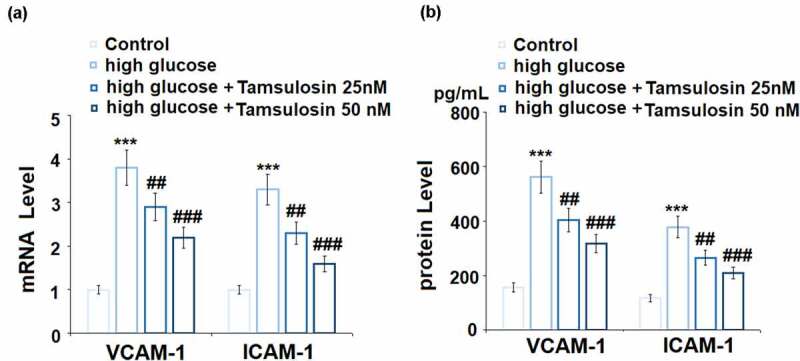
Tamsulosin prevented high glucose-induced expression of VCAM-1 and ICAM-1 in human glomerular endothelial cells (GECs). (a). mRNA of VCAM-1 and ICAM-1; (b). Protein of VCAM-1 and ICAM-1 (***, P < 0.005 vs. vehicle group; ##, ###, P < 0.05, 0.01 vs. high glucose group, N = 4)

The results in Figure 4a show that high glucose upregulated the mRNA levels of VCAM-1 and ICAM-1 to 3.8 – and 3.3 – fold, respectively, which were reduced to 2.9 – and 2.3 – fold by 25 nM Tamsulosin, respectively. 50 nM Tamsulosin further reduced the mRNA levels of VCAM-1 and ICAM-1 to 2.2 – and 1.6 – fold, respectively. Congruously, Tamsulosin downregulated the protein levels of VCAM-1 and ICAM-1 induced by high glucose (Figure 4b). Our results indicate that Tamsulosin could suppress the overexpression of adhesion molecules in high glucose-challenged GECs.

### 3.4 Tamsulosin inhibited high glucose-induced expression of fibrosis factors in human GECs.

**Figure 5.**
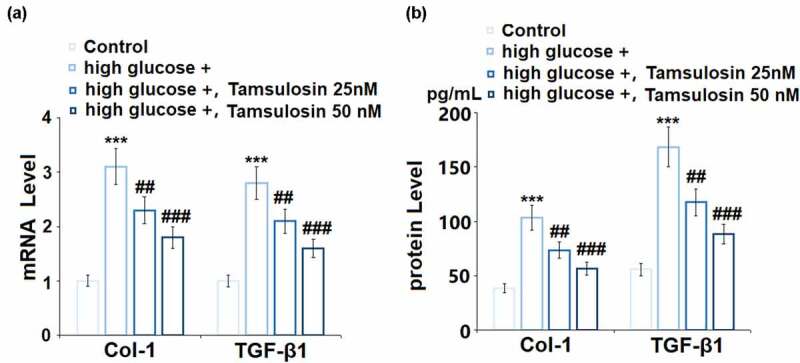
Tamsulosin inhibited high glucose-induced expression of fibrosis factors. (a). mRNA of Col-1 and TGF-β1; (b). Protein levels of Col-1 and TGF-β1 (***, P < 0.005 vs. vehicle group; ##, ###, P < 0.05, 0.01 vs. high glucose group, N = 4)

Renal interstitial fibrosis plays a critical role in the development of DN, so we further investigated the effects of Tamsulosin on high glucose-induced expressions of Col-1 and TGF-β1. As expected, high glucose stimulation induced a 3.1 – and 2.8 – fold increase of Col-1 and TGF-β1 at the mRNA level, respectively. 25 and 50 Tamsulosin decreased these levels to 2.3 – and 1.8 – fold of Col-1, and 2.1 – and 1.6 – fold of TGF-β1, respectively (Figure 5a). Similarly, results in Figure 5b show that Tamsulosin suppressed the protein levels of Col-1 and TGF-β1 in a dose-dependent manner.

### 3.5 Tamsulosin ameliorated high glucose-induced production of mitochondrial ROS and activation of p38.

**Figure 6.**
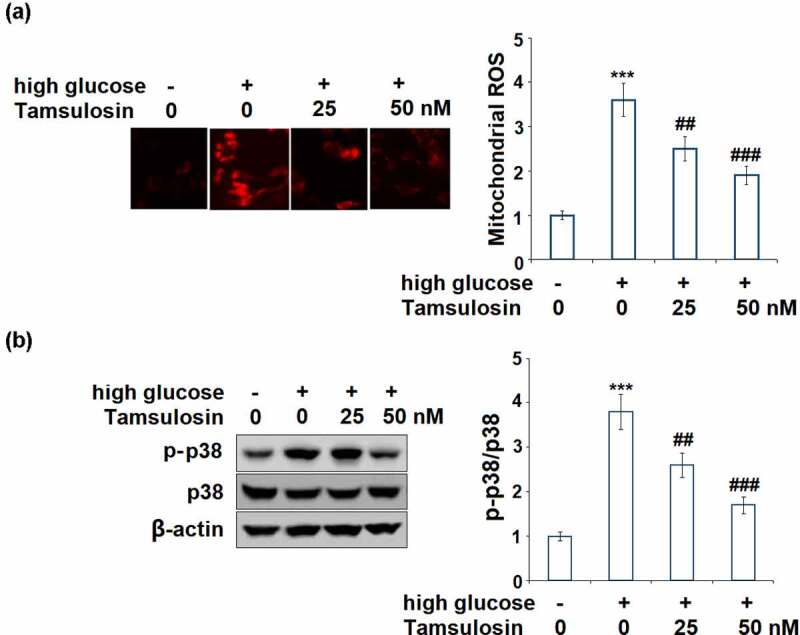
Tamsulosin ameliorated high glucose-induced production of mitochondrial ROS and activation of p38. (a). Mitochondrial ROS; (b). Levels of phosphorylated p38 and total p38 were measured (***, P < 0.005 vs. vehicle group; ##, ###, P < 0.05, 0.01 vs. high glucose group, N = 4–5)

We then evaluated the effects of Tamsulosin on high glucose-induced production of mitochondrial ROS and activation of p38. 25 and 50 nM Tamsulosin reduced the production of mitochondrial ROS to 2.5 – and 1.9 – fold, respectively, compared to the 3.6 – fold increase induced by high glucose (Figure 6a). The same doses of Tamsulosin reduced the activation of p38 to 2.6 – and 1.7 – fold, respectively, compared to the 3.8 – fold increase induced by high glucose, respectively (Figure 6b).

### 3.6 Tamsulosin suppressed high glucose-induced activation of NF-κB.

**Figure 7.**
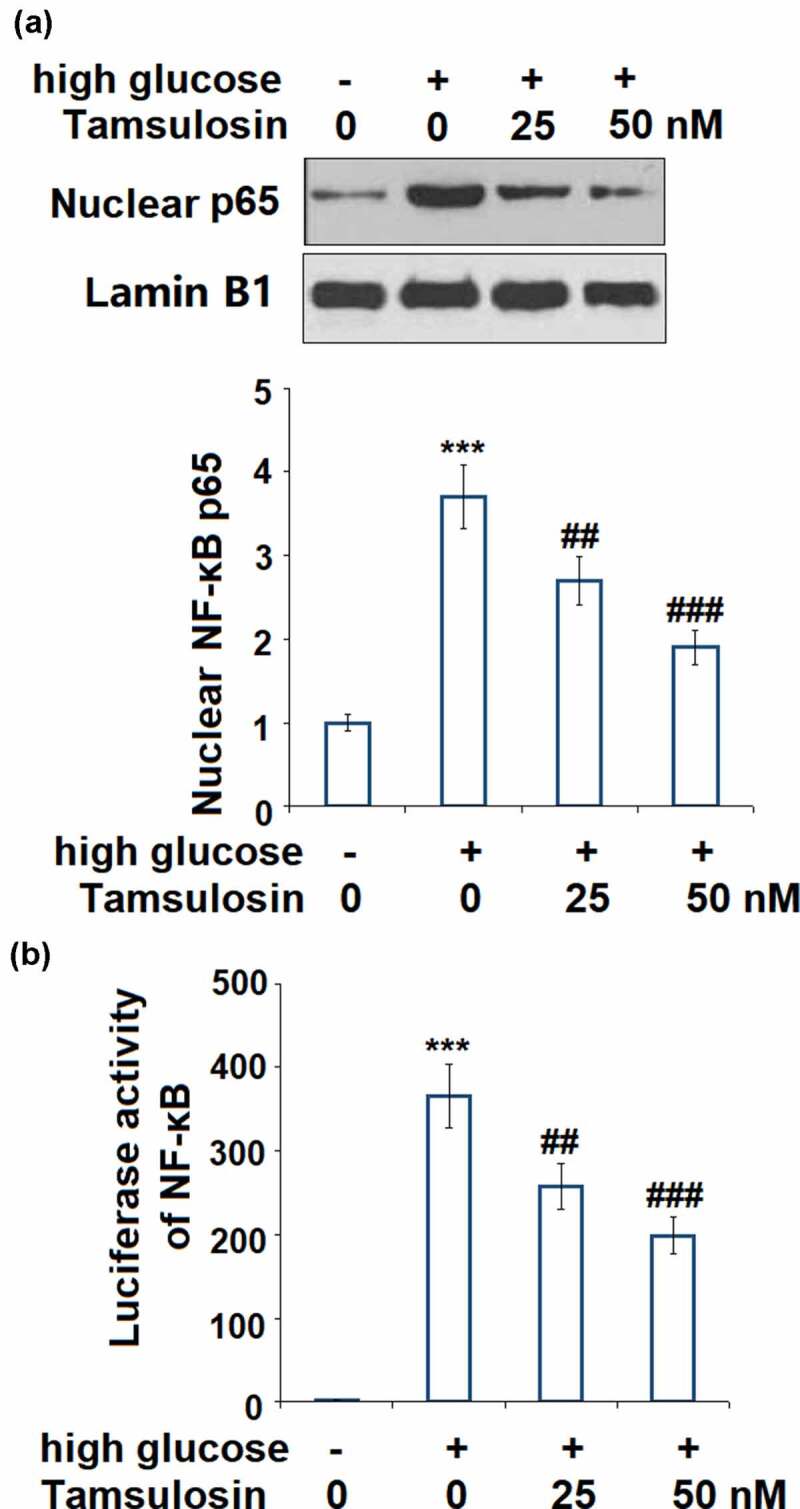
Tamsulosin suppressed high glucose-induced activation of NF-κB. (a). Nuclear level of NF-κB p65; (b). Luciferase activity of NF-κB (***, P < 0.005 vs. vehicle group; ##, ###, P < 0.05, 0.01 vs. high glucose group, N = 5)

To further verify the underlying mechanism of Tamsulosin on high glucose-challenged GECs, we investigated the activation of NF-κB. High glucose stimulation significantly upregulated nuclear NF-κB p65 to 3.7 – fold, which was decreased to 2.7 – and 1.9 – fold by 25 and 50 nM Tamsulosin, respectively (Figure 7a). Correspondingly, the same doses of Tamsulosin reduced the luciferase activity of NF-κB promoter to 257.8 – and 198.9 – fold, respectively, compared to the 365.6 – fold increase induced by high glucose (Figure 7b).

### 3.7. Tamsulosin ameliorated high glucose-induced reduction of nitric oxide (NO)

NO has been considered a pivotal vasoprotective molecule and plays an essential role in maintaining the normal function of endothelial cells. The decreased production of NO is reported in pathological states, which results in serious problems in endothelial equilibrium. Here, we found that exposure to high glucose significantly decreased the levels of NO, which were rescued by 25 and 50 nM Tamsulosin in a dose-dependent manner (Supplementary Figure 1). These findings suggest that Tamsulosin could maintain the endothelial function of GECs against high glucose.

## 4 Discussion

DN is a common complication of diabetes mellitus and is considered a major cause of diabetes-related death [17]. Studies have demonstrated that high glucose plays a critical role in the initiation of DN [8,18]. GECs constitute the first layer of the glomerular filtration barrier and their injury is the main cause of structural changes to the glomerulus in early DN. Therefore, prevention of GECs injury is imperative for the treatment of DN. Here, we studied the beneficial effects of Tamsulosin on high glucose-induced injury in GECs.

The essence of DN is renal fibrosis. Oxidative stress has been implicated for its initiation, inflammatory response as its central event, and various signaling pathways and abnormal regulation of molecules promote the development of DN. Oxidative stress is defined as the imbalance between the oxidative and the anti-oxidative system due to excessive production of ROS, which can cause the dysfunction of GECs. The generation of ROS was significantly increased under high glucose conditions, resulting in damage to the renal interstitium, and eventually to renal angiosclerosis. Excessive ROS also changes the function of the glomerular capillary wall leading to increased albumin permeability, which is responsible for the occurrence of microalbuminuria [19,20]. Furthermore, increasing studies have shown that overproduction of ROS can activate downstream modulators including p38 MAPK and NF-κB in GECs [21], triggering the cascade actions of various cellular responses which are responsible for the development of DN. Oxidative stress has become a promising target for the treatment of DN. In this study, we demonstrated that Tamsulosin successfully reduced the over-generation of ROS induced by high glucose in GECs.

Inflammation plays a critical role in the progression and development of DN. Its effects on damages in kidney tissues give rise to glomerular sclerosis and interstitial fibrosis [22]. High glucose induces an inflammatory cascade and enhances the expressions of adhesion molecules in GECs. TNF-α is an important pro-inflammatory cytokine involved in various inflammatory responses [23–25]. Previous studies have reported that the TNF-α level in patients with DN is higher than in healthy people [26]. TNF-α is cytotoxic to GECs and can directly induce apoptosis [27]. Enhanced expression of TNF-α induced by high glucose. in turn, induces the expressions of other inflammatory factors, including IL-6 and IL-8. The functions of IL-6 in kidney diseases have been well studied, its involvement and pro-inflammatory actions enhance endothelial permeability, resulting in microalbuminuria in early DN [28]. IL-8 is the pro-inflammatory chemokine responsible for the recruitment and activation of immune cells to injured endothelium [29]. The interaction of these pro-inflammatory factors leads to the development of DN. Furthermore, we found that high glucose-induced the expressions of ICAM-1 and VCAM-1, which promote the inflammatory responses in DN. We assessed the anti-inflammatory effects of Tamsulosin in high glucose-challenged GECs and as expected, Tamsulosin successfully reduced the expressions of TNF-α, IL-6, IL-8, ICAM-1, and VCAM-1, suggesting a robust anti-inflammatory property. Metalloproteinases (MMPs) are the most important molecules responsible for renal failure. MMP-2 and MMP-9 take part in the destruction of the glomerular basement membrane, renal inflammation, and fibrosis [30]. Overexpression of MMP-2 results in structural changes in the tubular basement membrane and can generate all of the common features of kidney disease, especially glomerulosclerosis and interstitial fibrosis [31]. MMP-9 is directly connected with the degree of proteinuria and is recognized as a marker of kidney damage. It promotes the development of DN toward end-stage renal disease (ESRD). Furthermore, MMP-2 and MMP-9 directly affect renal tubular cells, especially by impairing the cell-to-cell adhesion, leading to epithelial-to-mesenchymal transition (EMT), which contributes to renal fibrosis. In the process of renal fibrosis, the involvement of TGF-β1 is indispensable. TGF-β1 is well known as an anti-inflammatory cytokine in the early stage of kidney injury [32]. Short-time activation of TGF-β1 promotes renal repair, however, continuous activation of TGF-β1 results in renal fibrosis [33]. TGF-β1 wields its pro-fibrotic effects through two pathways: Smad-dependent and – independent. In the Smad-dependent pathway, Smad2 and Smad3 are highly activated resulting in their translocation into the nucleus to govern the expressions of pro-fibrotic factors, such as collagens [34,35]. The Smad-independent pathway involves TGF-β1 activating various downstream pathways, such as TGF-β1/p38 MAPK [36], Akt/mTOR [37], Wnt/β-catenin [38], and so on. These pathways are largely responsible for the development of renal fibrosis [39–41]. In high glucose-challenged GECs, both mRNA and protein levels of type 1 collagen (Col-1) and TGF-β1 were highly increased but were reduced by the two doses of Tamsulosin, indicating its strong anti-fibrosis capacity.

To explore the underlying mechanisms of the inhibitory effects of Tamsulosin on pro-inflammatory factors, we further investigated its effect on the NF-κB signaling pathway. Normally, NF-κB exists in an inactive state in cells. It is activated by specific stimuli, and in turn, activates the downstream pathway [42]. Activation of NF-κB is enhanced in patients with DN, consistently, our findings showed that high glucose significantly increased the nuclear level of NF-κB. Inhibition of NF-κB can reduce inflammation to alleviate renal injury [43]. Excessive activation of NF-κB increases the production of pro-inflammatory cytokines, chemokines, cellular adhesion molecules, and other factors involved in the inflammatory response, including TNF-α, IL-6, IL-8, ICAM-1, VCAM-1, MMP-2 and MMP-9. Our data demonstrated that Tamsulosin reduced the activation of NF-κB induced by high glucose, suggesting a wide range of anti-inflammatory capacity of Tamsulosin.

The major limitation of the current study is that we only assessed the beneficial effects of Tamsulosin against high glucose in an *in vitro* primary GECs culture model. The pathophysiological mechanisms of DN are complex and elusive. A wide range of risk factors have been involved in the pathogenesis of DN, including genetics, aging, and obesity. Therefore, further investigations with animals or clinical trials are warranted to investigate the effectiveness of Tamsulosin in the prevention of DN.

## Conclusion

To sum up, we found that Tamsulosin can prevent GECs injury induced by high glucose by alleviating oxidative stress, inhibiting inflammation, and slowing down fibrotic processes. In the future, we will further investigate the protective benefits of Tamsulosin and provide new evidence to elaborate on the underlying mechanism of Tamsulosin in the development of DN.

## Supplementary Material

Supplemental Material
