# Supplementary figures and images for: Tamsulosin attenuates high glucose- induced injury in glomerular endothelial cells
Source: Bioengineered. 2021 Aug 17;12(1):5184–94. doi: 10.1080/21655979.2021.1955527 (PMC8806910; doi:10.1080/21655979.2021.1955527)

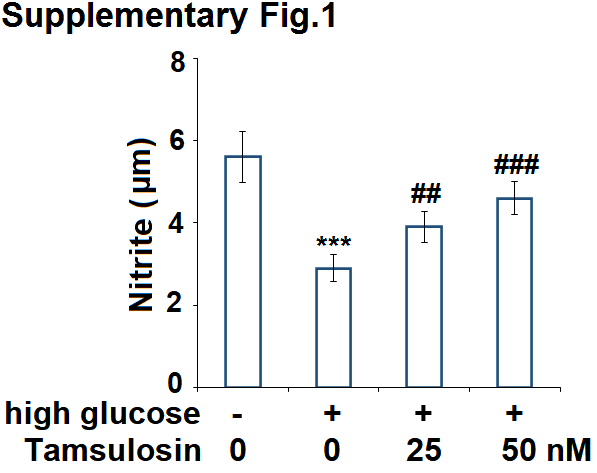

Supplement: Supplemental Material [file KBIE_A_1955527_SM7014.zip › supplementary Fig1.tif]
